# Associations of oral health status and swallowing function with cognitive impairment in the aging population: a cross-sectional study

**DOI:** 10.1186/s12903-023-03640-5

**Published:** 2023-11-23

**Authors:** Yong Chen, Canyang Li, Yongmei Fan, Lili Jiao, Matthew Silverman, Masashi Ishimaru, Jing Wang, Alice J. Van Pelt, Rumi Wang

**Affiliations:** 1 Laboratory of Food Oral Processing, School of Food Science & Biotechnology, Zhejiang Gongshang University, Hangzhou, Zhejiang China; 2 Rehabilitation Medicine Department, Speech and Language Pathology Therapy Section, The Second Xiangya Hospital of Central South University, Changsha, Hunan China; 3 Rehabilitation Medicine Department, The Second Xiangya Hospital of Central South University, Changsha, Hunan China; 4 Clinical Nursing Teaching and Research Section, The Second Xiangya Hospital, of Central South University, Changsha, Hunan China; 5 Department of Surgery, School of Medicine and Public Health, University of Wisconsin – Madison, Madison, Wisconsin USA; 6 PanasonicElectric Works Co, Ltd, Osaka Japan; 7 College of Biosystems Engineering and Food Science, Zhejiang University, Hangzhou, Zhejiang China; 8 Section of Gastroenterology, Jr. VA Hospital, Edward Hines, Hines, IL USA; 9 Division of Gastroenterology and Nutrition, Loyola University Stritch School of Medicine, Maywood, IL USA; 10 Rehabilitation Medicine Department, Speech and Language Pathology Therapy Section, The Second Xiangya Hospital of Central South University, Changsha, Hunan China

**Keywords:** Oral health, Swallowing function, Cognitive impairment, Older adults, Cross-sectional study

## Abstract

**Background:**

The purpose of this study was to investigate the relationships of oral health status and swallowing function with cognitive impairment in community-dwelling older adults from Changsha, Hunan Province, China.

**Methods:**

In this cross-sectional study, we analyzed the data of 215 participants aged ≥ 50 years which were retrieved from the Xiangya and Panasonic mild cognitive impairment (MCI) Study, a community-based study conducted among the residents of the urban areas of Hunan province in China. Demographic information of all participants was collected. We determined oral function by evaluating oral hygiene, oral dryness, occlusal force, tongue pressure, chewing function, swallowing function, remaining teeth number, and other indicators. The mini-mental state examination (MMSE) was used to screen for cognitive function. The relationship between each oral function evaluation item and cognitive function was investigated using correlation analysis. The associations between oral health status and swallowing function with cognitive impairment were inferred using multiple regression analysis.

**Results:**

The general characteristics of participants showed statistically significant correlation coefficients in number of teeth remaining (*p* = 0.003) and number of teeth lost (*p* < 0.0001). Almost half of the 25 participants (48%) were aged from 70–80 years. Only 25 older adults (11.6% of the participants) were determined to have cognitive impairment by MMSE sores less than 24. Tongue pressure in male participants was the only significant independent variable that was associated with cognitive impairment (*p* = 0.01971). The results indicate that male participants with lower MMSE scores had a relative deficiency in tongue pressure.

**Conclusions:**

In this cross-sectional study, the oral health status and swallowing function of participants were in relatively good condition and showed low correlations with cognitive impairment. However, lower tongue pressures were associated with lower MMSE scores in males, indicating it could serve as a novel oral function index for evaluating cognitive impairment.

## Background

Cognitive impairment in the elderly has become a very important social and medical problem in many countries with large aging populations. According to recent research, the global prevalence of mild cognitive impairment varies between 6.7% and 25.2% of people over 60 [1]. Cognitive impairment is currently considered a precursor to Alzheimer's disease. Prior studies found that 10%-15% of patients with mild cognitive impairment develop Alzheimer's disease every year. Therefore, early screening and interventions for patients with mild cognitive impairment are important to prevent and delay the development of Alzheimer's disease [2]. However, it is challenging to successfully predict and diagnose mild cognitive impairment, and many patients who develop Alzheimer's disease remain underdiagnosed in its early stages.

To better predict cognitive impairment in elderly patients, researchers have performed several studies which focus on the relationship between oral health and cognition in the elderly [3–6]. Poor oral health status is highly prevalent among the elderly with 95% of elderly patients suffering from oral diseases and is likely higher in senile dementia patients [7, 8]. It is still unclear how oral cavity changes in the elderly, such as a thicker tongue coating and higher degree of microorganism adhesion, lead to higher rates of oral infection, aspiration pneumonia, and postoperative infections [9–12]. Dry mouth and decreased saliva secretion contribute to denture incompatibility, taste disorders, aspiration pneumonia, difficulties with food intake, swallowing, and speaking [13–15]. When saliva secretion decreases and the saliva flow rate is lower than the absorption rate of oral mucosa fluid and the evaporation rate of oral fluid, the patient is more likely to be diagnosed with both insufficient saliva secretion and secondary xerostomia [16, 17].

The loss of functional teeth leads to deterioration and weakness of chewing ability [18, 19] and higher risk of dementia [20, 21]. A positive correlation between dementia and the number of missing teeth has been reported in the Chinese elderly population [22]. Some studies have found that premature and long-term tooth loss may be one of the causes of cognitive impairment in the elderly, so preventing these could be necessary to prevent cognitive decline. It is also reported that chewing exercises increase blood supply to the brain, which can also affect the learning and memory abilities of the brain [23, 24].

The decline of chewing ability affects the function and morphology of hippocampal neurons, which is very important for learning functions [18, 25–27]. Chewing can increase arousal, alertness, and motor control, which may improve cognitive ability. Lower occlusal force is related to declining chewing ability, and lower tongue pressure may affect normal chewing, bolus formation, and swallowing function, which may lead to malnutrition [28–30]. One study found that the relationship between maximum occlusal force and cognitive function can be directly or indirectly judged by dietary intake [31]. As tongue pressure and occlusal force are essential for nutrition intake, it may be important to assess tongue pressure as an early marker of mild cognitive impairment.

Comprehensive studies assessing oral health status using parameters such as tongue pressure, chewing, and swallowing, related to cognitive function are rare. Determining factors which are associated with cognitive decline can improve future patient outcomes by identifying potential treatment areas. Therefore, the purpose of this study is to explore the relationship among oral health status, swallowing function, and cognitive impairment of the elderly in the community of Changsha in China, and to determine if any parameters can predict cognitive decline.

## Methods

### Study design and population

This study was conducted in three communities in Changsha, Hunan province of China, and a total of 225 participants were recruited and 215 of them finished all the tests. Ten participants, who did not finish all the tests, were excluded. The sample size was set by using Student's t test (*n* = 22.0211) to determine the minimum sample size for each group, and 35 participants for each group were set as the minimum sample size in in this study. According to the three age groups (50–59 years, 60–69 years and 70–80 years) and with the ratio of male to female 1:1, a total of six groups and at least 210 participants were required. Finally, all data from 215 participants were collected by cross-sectional analysis in a singular evaluation. Participants ranged from 50 to 80 years old, and only included healthy adults who spoke Mandarin, understood the test steps and contents, and had no history of diagnosed neurological diseases. Individuals with diabetes, stroke, dysphagia, Parkinson's disease, or cognitive impairment were excluded from the study. All participants were lived in a fixed geographical area, such as ShiZiLing Community, YaoLing, and Weng Yi New Village in the Furong District of Changsha City.

All the processes, including recruitment, testing and data collection, were implemented during October to November 2021. Demographic information of all participants included age, gender, marital status, education level, occupation, family income, smoking, drinking, physical exercise, hobbies, life events, and past medical history. According to the criterion descried above, only eligible seniors were invited to participate the formal experiment. The details on the data collection procedure were showed as follows. In the first stage of data collection, the general information questionnaire, MMSE scale and EAT-10 scale questionnaire were collected for all participants. In the second stage, oral health examinations were performed to determine oral hygiene, oral dryness, occlusal force, tongue pressure, chewing function, and the number of remaining teeth. All testing procedures were performed by trained staff to ensure the accuracy of the experimental data collection process. Meanwhile, in the whole process of data collection, strict quality control was carried out to ensure that the obtained data were accurate.

### Oral health examination

Oral health examinations were conducted by two trained staff to assess the number of teeth visually. The number of teeth lost, presence of dentures, and number of remaining natural teeth were recorded for each participant [32]. Residual teeth and teeth with looseness of more than 3 degrees were not included. Participants were categorized based on the number of remaining natural teeth: less than 10 teeth, 10–19 teeth, and more than 20 natural teeth. This classification was chosen because previous studies used 20 or more existing natural teeth as fully functional dentition [3, 33, 34]. It has been reported that having less than 10 remaining teeth is an independent risk factor for cognitive impairment [35]. The loss of more than 16 teeth among the Chinese elderly population has been associated with severe cognitive impairment [22]. Therefore, our study uses the same standard for comparative analysis as with similar studies [36].

### Oral hygiene

Trained staff evaluated oral hygiene by observing the degree of tongue coating adhesion [9]. The tongue surface was divided into nine areas, and the degree of tongue coating adhesion in each area was scored in 3 sections. Score 0: The tongue coating is not visible; Score 1: the tongue coating is thin, and the nipple of tongue is visible; Score 2: the tongue coating is too thick that the nipple of the tongue is not visible. Tongue coating index (TCI) was calculated by the tongue surface score. Tongue coating adhesion degree above 50% was considered unhygienic [10].

### Oral dryness

We used the Saxon test to measure oral saliva levels and evaluate oral dryness [37, 38]. Medical gauze (Type III medical gauze piece, 7.5 cm, 12ply, dry weight 2 g) was put into the oral cavity of the participants. The participants were instructed to close their lips, and not swallow while chewing for 2 min. The gauze was then removed and placed into a cup for measurement. The amount of saliva was calculated by measuring the weight change of the gauze before and after chewing. When the weight of gauze increased by < 2 g after the Saxon test, the participants were classified as having oral dryness.

### Tongue pressure

Tongue pressure was measured by trained staff using a tongue pressure TPS100(Henan Xiangyu Medical equipment Co., LTD, China) [39, 40]. For denture wearers, tongue pressure was measured when installing the denture. A sublingual airbag was installed on the tongue pressor (TPS100). Staff demonstrated placement of the sublingual airbag to the participants using an oral model, with the front teeth pressed against the thick and thin interfaces of the sublingual airbag. After the participant placed the airbag in position, the staff reminded the participant to listen to the instructions. The participants pushed the balloon to the hard palate with the greatest strength of their tongue, and repeated three times in each round, for a total of three rounds. When the measured maximum tongue pressure was less than 30 kPa, the participants were classified as low tongue pressure.

### Occlusal force

The evaluation of occlusal force was based on the method described by Miyaura, which is replaced by an alternative examination [41], such as evaluating the number of remaining teeth and checking the grip strength visually. The evaluation method of the number of remaining teeth was the same as that in 2.2 Oral Health Testing. Subjects with less than 20 teeth after removing residual root teeth and teeth with looseness were classified as having a lower occlusal force [42].

### Swallowing function

The swallowing function was measured by trained staff using the swallowing dysfunction screening scale (EAT-10) [43]. It includes ten items, with scores ranging from zero to four: zero is no dysfunction, one is mild, two is moderate, three is severe, and four is most severe. When the score value of each item was three or higher, it was considered that the participant has an increased risk of dysphagia. When the total score of EAT-10 is ≥ 1, the sensitivity and negative predictive value are the best, and it can predict dysphagia, impaired swallowing ability, infiltration, and aspiration in patients with acute strokes. Trained staff used gelatinous fudge, put a gummy candy on the preferred side of participants, and guided them to chew with the greatest strength and fastest speed for 20 s [24, 44]. The participants were informed not to swallow saliva and candy, nor to change sides to chew. After stopping the time, they put 30 mL saline into their mouth and gargled five times, then spit it into a cup with a filter. Researchers then measured the amount of glucose dissolved using a blood glucose meter (GT-1970) to check the chewing efficiency. When the tested glucose concentration was less than 100 mg/dl, the chewing ability was considered impaired.

### Cognitive function assessment

Cognitive function was assessed by trained staff using the mini-mental state examination (MMSE-2) [45], which includes evaluating direction, attention, memory, language, and the ability to follow simple commands. MMSE scores range from 0 to 30, with higher scores indicating better cognition. Participants with a total score of less than 24 are defined as having "cognitive impairment". However, among people with different educational levels, the criterion of MMSE is different. In a study of the elderly in the community environment, the sensitivity of MMSE critical value 24 to normal cognition is 0.85, the specificity is 0.90, and the sensitivity to cognitive impairment is less than 24. People with MMSE scores higher than this critical value are classified as "normal cognition". All the examiners were blinded to the conditions that the participants were in only if applicable.

### Statistical analysis

The data collected in this study were analyzed using Python (3.7.3version, The Python Software Foundation), and the significance level was *p* < 0.05. We used descriptive statistics to report demographic characteristics. A Chi-square test was used to evaluate the differences in age, BMI, education level, foreign language ability, symptoms of dry mouth, difficulty in chewing food, reduction of food intake, number of teeth remaining, number of lost teeth, number of dentures, number of brushing teeth, etc. between the normal group and the cognitive impairment group. To explore the correlation between related factors of oral function and cognitive function, linear regression analysis was used to investigate the factors influencing cognitive impairment.

## Results

### General characteristics of the study participants

The general characteristics of the participants were classified into two groups according to their MMSE scores and presented in Table 1. In total, 215 older Chinese adults from Changsha participated in this cross-sectional study. The age of the participants ranged from 50–80 years and the gender ratio of male and female was approximately 1:1. Only 25 older adults (11.6% of the participants) were identified as having cognitive impairment with MMSE scores less than 24. Almost half of the 25 participants (48%) were 70–80 years old. There was no significant difference in the distributions of BMI (*p* = 0.077) and 89.3% of the participants ranged from BMI of 18–28. The education levels of the participants were divided into three groups based on highest level completed, including primary school, middle school, and university. There was a significant difference between the three levels with *p*-value < 0.001. For the cognitively impaired participants, most of them obtained a middle school level of education or less.

**Table 1:** General characteristics of study subjects according to cognitive function (*N* = 215)

	Normal (MMSE^a^≧24)		Cognitive Impairment (MMSE^a^ < 24)		Total		x^2^	*p*-value
Age								
50–59	64	33.7%	7	28.0%	71	33.0%	3.1802	0.2039
60–69	68	35.8%	6	24.0%	74	34.4%		
70–80	58	30.5%	12	48.0%	70	32.6%		
Gender								
Male	96	50.5%	12	48.0%	108	50.2%	0.0006	0.9803
Female	94	49.5%	13	52.0%	107	49.8%		
BMI								
< 18	1	0.5%	0	0.0%	1	0.5%	6.8315	0.0775
18–24	88	46.3%	12	48.0%	100	46.5%		
24–28	85	44.7%	7	28.0%	92	42.8%		
> 28	16	8.4%	6	24.0%	22	10.2%		
Education								
Primary school	14	7.4%	9	36.0%	23	10.7%	20.7207	< 0.001**
Middle school	136	71.6%	15	60.0%	151	70.2%		
University	40	21.1%	1	4.0%	41	19.1%		
Self-reported Dry Mouth								
Yes	39	20.5%	7	28.0%	46	21.4%	0.3567	0.5504
No	151	79.5%	18	72.0%	169	78.6%		
Self-reported Chewing Difficulty								
Yes	64	33.7%	10	40.0%	74	34.4%	0.1608	0.6885
No	126	66.3%	15	60.0%	141	65.6%		
Self-reported Food Intake Reduction								
Yes	49	25.8%	7	28.0%	56	26.0%	0.0000	0.9955
No	141	74.2%	18	72.0%	159	74.0%		
Number of Teeth								
< 10	0	0.0%	1	4.0%	1	0.5%	11.7637	< 0.001**
10–19	3	1.6%	2	8.0%	5	2.3%		
≧20	187	98.4%	22	88.0%	209	97.2%		
Number of Teeth Loss								
< 5	173	91.1%	18	72.0%	191	88.8%	30.9294	< 0.001**
5–8	16	8.4%	2	8.0%	18	8.4%		
≧9	1	0.5%	5	20.0%	6	2.8%		
Number of False Teeth								
< 10	176	92.6%	25	100.0%	201	93.5%	1.9704	0.3734
10–19	11	5.8%	0	0.0%	11	5.1%		
≧20	3	1.6%	0	0.0%	3	1.4%		
Tooth-brushing Frequency								
< 2	29	15.3%	3	12.0%	32	14.9%	0.0174	0.8949
≧2	161	84.7%	22	88.0%	183	85.1%		

^**^P-value < 0.001 means there are significant associations between the corresponding characteristics(including Education,Number of teeth and Number of teeth loss) and cognitive function

^a^ MMSE, mini-mental state examination

### Self-reported questionnaires

There were no significant differences in dry mouth, chewing difficulty, food intake reduction, number of false teeth, or tooth-brushing frequency between the normal group and cognitively impaired group. In both groups, less than 40% of participants reported problems with dry mouth, chewing difficulty, and food intake reduction. There were significant differences in number of teeth remaining (*p* = 0.003) and number of teeth lost (*p* < 0.001). Significantly, male participants with more teeth were found to have higher MMSE scores. The results also showed that 97.2% of all participants had at least 20 teeth. In general, all the results obtained by self-reported questionnaires indicated the majority of the older adults in this study considered their oral health to be in relatively good condition.

### Cognitive impairment and oral health

**Fig. 1:**
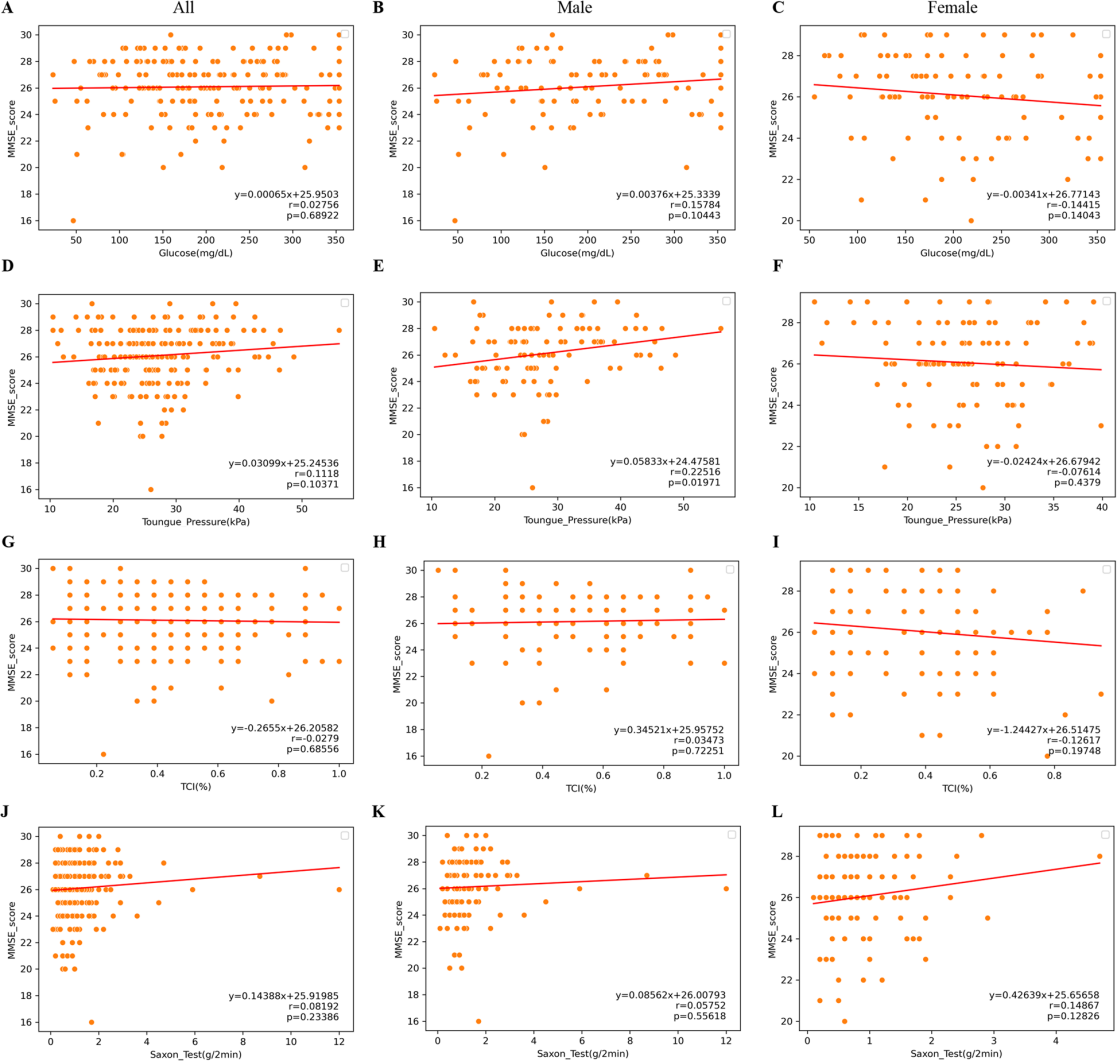
The associations between oral functions and cognitive impairment

The associations of oral health condition with cognitive impairment were analyzed and the correlation results are shown in Fig. 1. The occlusal force measured by glucose test indicated that the correlations between MMSE scores and occlusal force in all participants (Fig. 1A), male (Fig. 1B) and female participants (Fig. 1C) showed no significant differences with *p*-values of 0.68922, 0.10443 and 0.14043, respectively. The results of tongue pressure showed that the correlations between MMSE scores and tongue pressure in all participants (Fig. 1D) and female participants (Fig. 1F) showed no significant differences with *p*-values of 0.10371 and 0.4379, respectively. However, in male participants (Fig. 1E), the correlations showed strong differences with *p*-value = 0.01971. Moreover, the oral hygiene measured by the TCI test indicated that the correlations between MMSE scores and TCI% in all participants (Fig. 1G), male (Fig. 1H) and female participants (Fig. 1I) showed no significant differences with *p*-values of 0.68556, 0.72251 and 0.19748, respectively. Additionally, the oral dryness measured by Saxon test indicated that the correlations between MMSE scores and oral dryness degree in all participants (Fig. 1J), male (Fig. 1K) and female participants (Fig. 1L) showed no significant differences with *p*-values of 0.23386, 0.55618 and 0.12826, respectively.

### Cognitive impairment and swallowing function

**Fig. 2:**
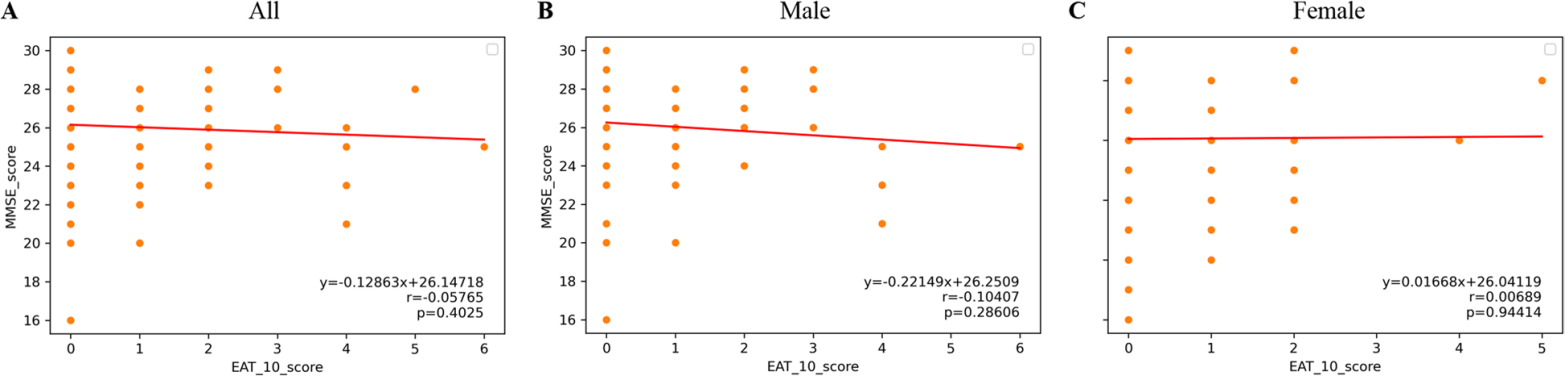
The associations between swallowing function and cognitive impairment

The swallowing function evaluated by EAT-10 indicated that the correlations between MMSE and EAT-10 scores in all participants (Fig. 2A), male (Fig. 2B) and female participants (Fig. 2C) showed no significant differences with *p*-values of 0.4025, 0.28606 and 0.94414, respectively.

## Discussion

This study explored the associations of oral health status and swallowing function in patients with normal to mild cognitive impairment. The general characteristics of participants showed that there were statistically significant correlation coefficients in number of teeth remaining and number of teeth lost. Tongue pressure in male participants was the only significant independent variable among all being studied that was associated with cognitive impairment. The results indicated that the male participants with lower MMSE scores had a relative deficiency in tongue pressure, which could be used as an easily evaluated, novel predictor for cognitive impairment evaluation.

The general characteristics of the sample were critical for explaining the results different from other studies. In recent years, many studies evaluating the oral function of older adults with mild cognitive impairment (MCI) or dementia have demonstrated that various factors (e.g. age, education levels, number of teeth and frailty, marital status, and use of dental prostheses) are significantly associated with MCI [22, 46]. A previous study including 3063 older adults in Shanghai showed that participants with dementia had older age, less years of education, and lower MMSE scores when compared to the cognitively normal older adults [22]. In our study, only 215 older Chinese adults from Changsha participated, with a mean age of 64.7 years. We also found the subjects with mild cognitive impairment were older on average by showing that almost half of them were aged from 70–80 years. However, there was no significant difference in the association of age and education levels with MCI between the normal group and cognitive impairment group. We also found no correlations between the control group and the cognitive impairment group in dry mouth, chewing difficulty, food intake reduction, number of false teeth, and tooth-brushing frequency. Most of the participants suggested that they were in relatively good health condition by self-evaluation. Our study’s smaller population size of only 215 participants and younger mean age of the elderly, may explain differences from other studies.

Previous studies have reported that cognitive impairment is generally associated with oral health functions, including oral hygiene, occlusal force, and orofacial pain [47–51], etc. A study suggested that deficient daily oral hygiene was closely associated with MCI even at an early stage [51]. However, in this study, the results did not show great differences between oral hygiene and MCI. Moreover, it was reported that in the older adults with retained competence, maximal occlusal force was frequently present in participants with MCI or dementia. The occlusal force measured by glucose test also showed no correlations with MCI. Interestingly, lower tongue pressure in male participants showed strong correlation with MCI. To the best of our knowledge, this paper is the first to indicate tongue pressure as a possible predictor for evaluating MCI. Recently, a 2-year follow-up study indicated that tongue pressure was significantly stronger in the healthy group than in the cognitive impairment group [52]. In addition, the study suggested that improvements in the MMSE score were correlated with an increase in both tongue pressure and masticatory performance. In another recent study, orofacial pain and its potential causes were statistically associated with MCI, which could be another potential indicator for early diagnosis of MCI. In addition, a previous study suggested that participants having markedly fewer occluding pairs and smaller active mouth openings had more severe cognitive impairment [49].

A prospective cohort study of older adults in intermediate care in Sweden identified swallowing dysfunction and poor oral health as independent risk factors for mortality [53]. A very recent systematic review and meta-analysis of 1427 participants showed that some cognitive disorders were significantly associated with dysphagia [54]. Moreover, the association between cognition and swallowing disorders indicates that multiple neuroanatomical systems might be involved in these two functions. In contrast, the results in the present study showed no statistical correlations between swallowing function and MCI. For most of the participants, their swallowing functions were perceived as strong. The oral health status and swallowing ability were reported as good. Finally, the number of older adults in present study classified as having MCI was low, thus reducing the power of statistical tests to demonstrate associations. Nevertheless, the results still suggest that male participants with lower tongue pressure were at greater risk of MCI or dementia.

## Conclusions

The present study is the first to indicate tongue pressure as a possible predictor for evaluating mild cognitive impairment. However, cognitive impairment is influenced by complex relationships with oral health status and swallowing function. Novel and comprehensive evaluations by using interdisciplinary methods are needed for early diagnosis of mild cognitive impairment. Moreover, there were several limitations in present study, including the sample size, this specific populations, as well as the used statistical analysis. Further analysis on the associations of oral functions with mild cognitive impairment are still needed to confirm these findings in the future.

## Data Availability

The datasets analyzed in the current study are not publicly available due to the need for the Panasonic Electric Works Company to protect data privacy for further study, but are available from the corresponding author on request.

## Abbreviations

MCI: Mild cognitive impairment
MMSE: Mini-mental state examination
TCI: Tongue coating index
EAT-10: The swallowing dysfunction screening scale
